# Parental Acceptance, Parental Hesitancy, and Uptake of Seasonal Influenza Vaccination among Children Aged 6–59 Months: A Systematic Review and Meta-Analysis

**DOI:** 10.3390/vaccines11081360

**Published:** 2023-08-13

**Authors:** Paul Shing-fong Chan, Yuan Fang, Joseph Kawuki, Siyu Chen, Xue Liang, Phoenix Kit-han Mo, Zixin Wang

**Affiliations:** 1 Centre for Health Behaviours Research, Jockey Club School of Public Health and Primary Care, The Chinese University of Hong Kong, Hong Kong, China; pchan@link.cuhk.edu.hk (P.S.-f.C.); joseks256@gmail.com (J.K.); chensiyu@link.cuhk.edu.hk (S.C.); 1155187741@link.cuhk.edu.hk (X.L.); phoenix.mo@cuhk.edu.hk (P.K.-h.M.); 2 Department of Health and Physical Education, The Education University of Hong Kong, Hong Kong, China; lunajoef@gmail.com

**Keywords:** children, parental acceptance, parental hesitancy, uptake, seasonal influenza vaccination

## Abstract

This systematic review and meta-analysis summarises the literature on parental acceptance, parental hesitancy, uptake, and the associated factors of seasonal influenza vaccination (SIV) among children aged 6–59 months. Studies were sourced from the following platforms: PubMed, Web of Science, MEDLINE, and EMBASE databases. A random-effects model was used to evaluate the prevalence and 95% confidence intervals (CI) of parental acceptance, parental hesitancy, and SIV uptake in the last flu season and lifetime among children. A total of 36 studies were included for analysis. The overall prevalence was 64% for parental acceptance (95% CI: 51–75%), 34% for parental hesitancy (95% CI: 22–48%), 41% for SIV uptake in the last flu season (95% CI: 33–50%), and 46% for SIV uptake in a lifetime (95% CI: 20–74%). Associated factors of parental acceptance/hesitancy and uptake included the age of the children or parents, parental education level, household income level, ethnicity, and other modifiable factors, including perceived benefits, perceived barriers, perceived severity, perceived susceptibility, and cues to action related to SIV. Meta-regression analyses revealed regional differences in parental acceptance (Americas: 79% vs. Asia: 60%). The results provided implications informing us of the development of intervention programs targeting parents to improve SIV coverage among young children.

## 1. Introduction

Seasonal influenza is a common respiratory tract infection and a public health problem, particularly in children [1]. It is a significant cause of illness in young children aged under five years, who are at a greater risk of complications, which results in an estimated 870,000 hospitalisations and 10,200 deaths across the globe every year [2,3,4,5]. Children aged 6–59 months are more susceptible to seasonal influenza and have more severe consequences than the older groups [6,7]. For instance, children aged 6–59 months had higher rates than those aged 5–59 years in infection (83.7 vs. 10.2–16.4 per 100,000 individuals), hospitalisation (6.7–10.9 vs. 1.7–3.2 per 10,000 individuals), and mortality of seasonal influenza (0.2 vs. 0.02–0.04 per 100,000 individuals) [6,7]. Therefore, effective measures, such as vaccination, to prevent younger children from seasonal influenza infection are of vital importance [6,7].

Seasonal influenza vaccination (SIV) is considered one of the most effective ways to prevent influenza illness and severe complications from influenza virus infection [4,8,9]. It was shown that vaccinated young children had fewer respiratory illnesses and primary care consultations than their unvaccinated counterparts, and SIV could help reduce hospitalisation by 70% [10,11]. It was reported that not only did SIV protect the children, but it also protected the whole community and helped reduce influenza incidence in the general population [12]. Due to the constant mutation of the influenza virus, the World Health Organization (WHO) recommends children aged 6–59 months receive SIV every year [13]. Many countries implemented this guideline for SIV among children aged 6–59 months [14,15,16].

Despite the WHO’s recommendations, the successful implementation of SIV for children faced various obstacles, resulting in a low immunisation rate. Since parents are the main healthcare decision-makers for their young children, parents’ attitudes and perceptions of SIV play an important role in children’s SIV uptake. Across countries, studies reported a higher level of parental hesitancy for SIV among parents of children under the age of five years than parents of children aged 5–18 years (43–85% vs. 19.5–25.8%) [17,18,19]. As a result, the SIV uptake among children aged 6–59 months in the last flu season in these countries (9–45%) was lower than their older counterparts (59%) as well as the target SIV coverage rate (75%) recommended by the WHO [13,20,21,22]. It is possible that the factors that influence parental decisions regarding the vaccination of young children may be different from those that influence the vaccination of older children. Parents of children aged under five years expressed concerns that it was too young for their children to receive SIV, and they worried that SIV would have a negative effect on interactions with other vaccines to be received by their children [23,24,25]. These parents also worried that their young children may catch influenza from the vaccine [26,27]. Furthermore, they believed that natural immunity to influenza was better for their young children [28]. All these are unique obstacles for parents to vaccinate their children aged under five years. A systematic review was conducted in 2017 to investigate factors affecting routine childhood vaccination, including SIV, among children aged under 5 years [29]. However, the aforementioned systematic review did not cover countries in which SIV was not part of routine childhood vaccination [30]. In many countries (e.g., China, Singapore, and Poland), routine childhood vaccination programs for children aged under 5 years did not include SIV. From the existing literature, it is observed that there is no systematic review specifically looking at SIV uptake among children aged under five years. Therefore, a thorough understanding of the level of parental acceptance, parental hesitancy, and SIV uptake among children aged under five years, as well as their associated factors, is important for intervention development and policymaking in order to increase SIV coverage in this group of children.

This systematic review and meta-analysis was, therefore, conducted to synthesise and summarise the available literature on the levels of parental acceptance, parental hesitancy, and SIV uptake in the last flu season and lifetime in children aged 6–59 months as well as factors associated with parental acceptance/hesitancy and children’s SIV uptake.

## 2. Materials and Methods

### 2.1. Study Design

This systematic review and meta-analysis was registered in PROSPERO (CRD42023428862) and conducted according to the PRISMA guideline [30]. We included studies reporting on parental acceptance, parental hesitancy, and uptake rates of SIV in the last flu season and lifetime among children aged 6–59 months. Studies were sourced from the following platforms: PubMed, Web of Science, MEDLINE, and EMBASE databases.

### 2.2. Search Strategy

A comprehensive search was conducted using a number of appropriate keywords in order to identify studies reporting on parental acceptance, parental hesitancy, uptake, and the associated factors of parental acceptance/hesitancy and children’s SIV uptake in children aged 6–59 months. To match the aim of this systematic review, there were five sets of keywords for searching articles, which were related to the following: (1) acceptance/hesitancy/uptake/determinants; (2) parent; (3) influenza; (4) vaccination; and (5) children. The research team discussed to achieve a consensus on the terms to be included in each set. The asterisk “*” was appended to each term to search for variations of those terms. Additionally, the Boolean operators “OR” was used to link each term in each set and “AND” was used to link each set of the terms. The complete search strategy is described as follows:

(1) willing* OR accept* OR refus* OR hesitan* OR inten* OR reject* OR declin* OR uptake* OR factor* OR determinant* OR reason* OR facilitator* OR barrier* OR attitude* OR perception* OR view* OR predict* OR enabl* OR knowledge OR perspective*;
(2) parent* OR guardian* OR caregiver*;
(3) influenza OR flu;
(4) vaccin* OR immuni*;
(5) child* OR infant* OR toddler* OR pediatr* OR preschool* OR kindergart*;
(6) 1 AND 2 AND 3 AND 4 AND 5.

### 2.3. Inclusion and Exclusion Criteria

Peer-reviewed original studies published in English-language journals using quantitative, qualitative, or mixed-method methodology were included in this systematic review. The eligible studies should report parental acceptance, parental hesitancy, uptake, and the associated factors of SIV in children aged 6–59 months. The articles were searched with no restriction on country or region. An article was excluded if it was (1) the development of a study protocol/scale/measurement tool; (2) unclear about whether the participants were parents/caregivers of children; (3) the modelling or projection for prediction of SIV uptake among children; or (4) other grey papers, such as government documents/reports, newspapers, textbooks, book chapters, and preprints. As government documents/reports and newspapers were not written for scientific purposes, they were excluded from consideration. In view of the fact that grey papers were generally published without peer review, there are concerns regarding the quality and reliability of these publications.

### 2.4. Data Extraction

Two independent reviewers (P.S.-f.C. and J.K.) carried out data extraction and reference searching parallelly. Five papers were randomly selected for initial data extraction. The two reviewers discussed the results of these five papers, and any discrepancies arising were resolved through consensus. After that, they continued to work on the data extraction of the rest of the papers. After finishing data extraction of all papers, they discussed all results, and consensus was achieved through discussions. A data extraction form was used, which included reference ID, first author, publication year, study year, title, country, study design, participants, sample size, parental acceptance or hesitancy rates, uptake rates in the previous flu season or lifetime among children aged 6–59 months, and other relevant findings on associated factors, attitude, and perception.

### 2.5. Quality Assessment

The information from the included articles was assessed using the Mixed-Method Appraisal Tool with detailed descriptions of the rating [31]. For quantitative studies, there are four assessment criteria: (1) whether the research question can be addressed using the sampling strategy; (2) whether the sample is representative of the population under study; (3) whether appropriate measurements are used, e.g., clear origin, reliability, and validity; (4) whether there is an acceptable response (at least 60%). For qualitative studies, there are also four assessment criteria: (1) whether the sources of qualitative data are relevant to address the research question; (2) whether the process for analysing qualitative data is relevant to address the research question; (3) whether appropriate consideration is given to how findings relate to the context, e.g., the setting, in which the data were collected; (4) whether appropriate consideration is given to how findings relate to researchers’ influence, e.g., via their interactions with participants. For mixed-method studies, in addition to the aforementioned criteria for the quantitative and qualitative studies, there are other three criteria: (1) whether the mixed-method research design is relevant to address the qualitative and quantitative research questions; (2) whether the integration of qualitative and quantitative data (or results) is relevant to address the research question; (3) whether appropriate consideration is given to the limitations associated with this integration, e.g., the divergence of qualitative and quantitative data. For each criterion, “Yes” or “No” is given as the rating [31]. Two authors (P.S.-f.C. and J.K.) independently assessed the quality of the included studies. Similar to data extraction, five papers were randomly selected for initial quality assessment. The two reviewers discussed the results of these five papers, and any discrepancies that arose were resolved through consensus. After that, they continued to work on the quality assessment of the rest of the papers. After finishing the quality assessment of all papers, they discussed all results, and consensus was achieved through discussions.

### 2.6. Statistical Analysis

Frequencies and percentages were used to summarise the characteristics of included studies. Random-effects models were used to generate the pooled uptake rates of children aged 6–59 months in the last flu season and lifetime, parental acceptance, and parental hesitancy. Q-test and *I*^2^ test were employed to assess heterogeneity among studies. The parental hesitancy rate was computed using the formula (100-parental acceptance rate) for studies that only provided information on the parental acceptance rate. Likewise, the parental acceptance rate was computed using the formula (100-parental hesitancy rate) for studies that only provided information on the parental hesitancy rate.

In order to determine if study characteristics could explain the variability between studies, subgroup analyses and meta-regression were conducted. This included study year (2010 or before, 2011–2015, 2016–2019, and 2020 or after), region (Asia, Americas, Europe, and Oceania), and sample size (≤1000 and >1000). In order to test whether the uptake of SIV in the last flu season and lifetime among children aged 6–59 months, parental acceptance, and parental hesitancy varied according to the selected study characteristics, univariate meta-regression was performed. Study characteristics were then included in the multivariable meta-regression if they were significant (*p* < 0.05) in the univariate analysis.

The presence of publication bias was visualised using funnel plots, and Egger’s linear regression test was performed. Meta-prop package (method = Inverse and summary measure = PLOGIT) was adopted in R Studio (version 4.2.1, https://cran.r-project.org/bin/windows/base/old/4.2.1/, accessed on 1 May 2023) to conduct the meta-analyses.

Additionally, other key findings on associated factors of parental acceptance, parental hesitancy, and uptake of SIV in the last season and lifetime among children aged 6–59 months, as well as parental attitudes and perceptions, were summarised thematically.

## 3. Results

### 3.1. Identification of Studies

**Figure 1:**
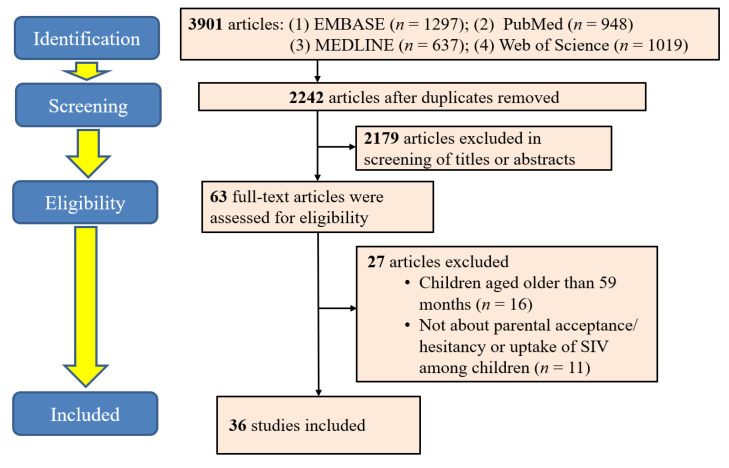
Flowchart of selection of included studies.

The initial search returned 3901 citations, and 2242 remained after excluding duplicates (Figure 1). After that, we further removed 2179 articles after screening for titles and abstracts. We performed full-text screening on 63 articles, of which 27 articles were excluded because they did not meet the selection criteria.

### 3.2. Overview of Included Studies

A total of 36 studies published from 2004 to 2023 were finally analysed and summarised [14,15,16,17,18,19,20,21,23,24,25,26,27,28,32,33,34,35,36,37,38,39,40,41,42,43,44,45,46,47,48,49,50,51,52,53] (Table 1). Most of the studies were performed in Asia (*n* = 15, 42%) and the Americas (*n* = 14, 39%), followed by Europe (*n* = 5, 14%) and Oceania (*n* = 2, 6%). These studies consist of 33 quantitative studies, 2 qualitative studies, and 1 mixed-method study. The total sample size is 68,567. Regarding study years, 14 studies were conducted before 2011, 9 were between 2011 and 2015, 10 were between 2016 and 2019, and 3 were in 2020 or after. Regarding the overall quality of the included studies, over 75% of them satisfied each criterion, except for the response rates in quantitative studies and issues related to researchers’ influence in qualitative studies. Less than half of the quantitative studies had a response rate higher than 60%. All qualitative studies did not have appropriate consideration given to how findings relate to researchers’ influence (Supplementary Table S1A–C).

### 3.3. Primary Findings

**Figure 2:**
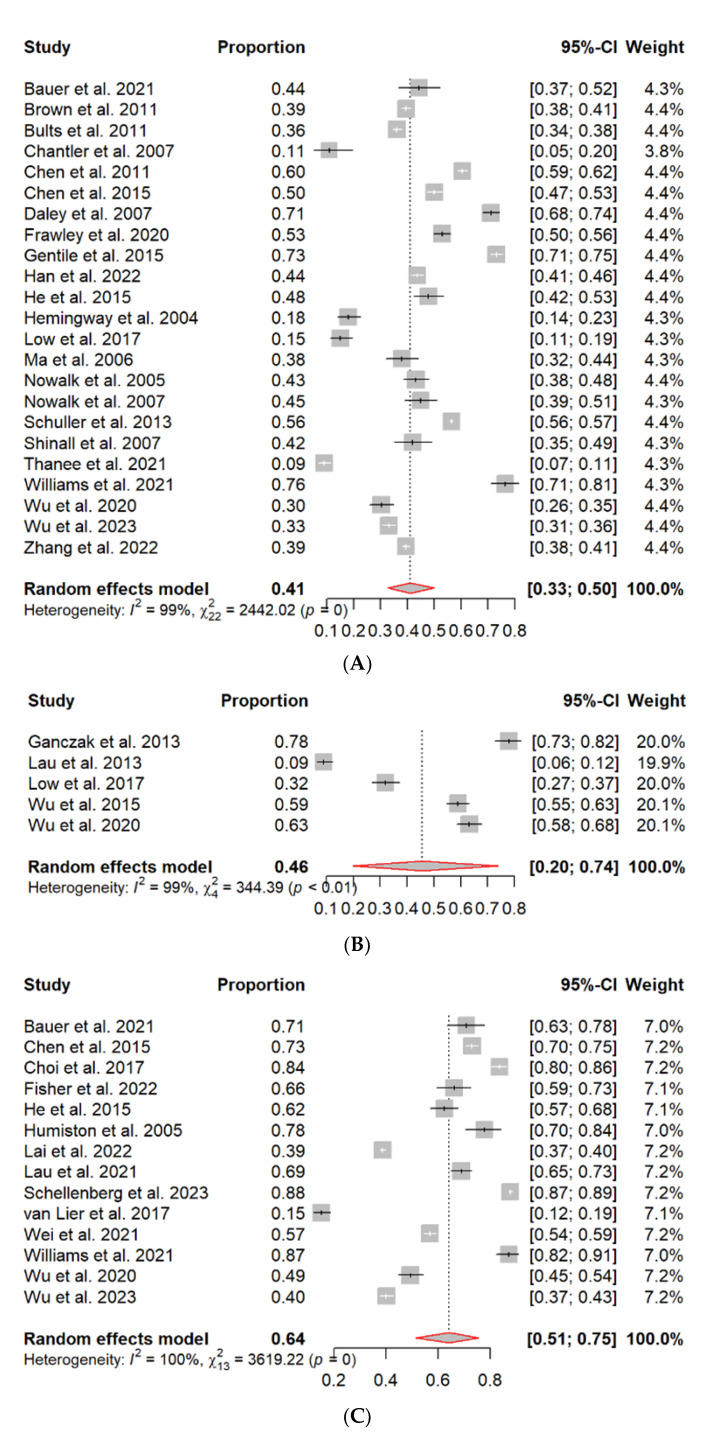

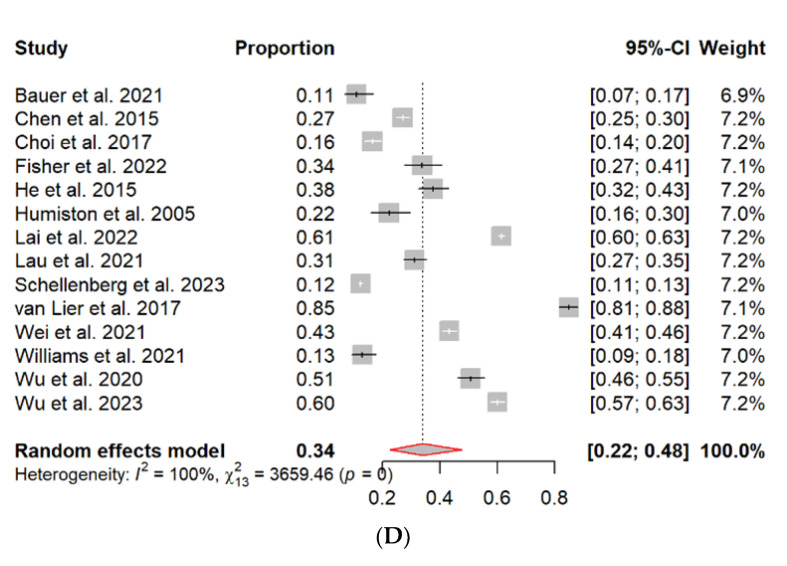
(**A**) Forest plot for pooled uptake rate of SIV in the last flu season; (**B**) forest plot for pooled uptake rate in lifetime; (**C**) forest plot for pooled acceptance rate of SIV; (**D**) forest plot for pooled hesitancy rate of SIV [14,15,16,17,18,19,20,21,23,24,25,26,28,32,33,34,35,36,37,38,39,40,41,42,43,44,45,46,47,48,50,51,52,53].

The pooled prevalence of parental acceptance, parental hesitancy, and the uptake of SIV in the last flu season and lifetime among children aged 6–59 months are presented in Figure 2A–D (forest plots), the associated factors are shown in Table 2, and parental attitudes and perceptions are presented in Table 1.

#### 3.3.1. SIV Uptake in the Last Flu Season among Children Aged 6–59 Months

Twenty-three studies reported on the SIV uptake in the last flu season (Figure 2A). The overall uptake rate was 41% (95% CI: 33–50%), with a range of 9% to 76%, and had a significant heterogeneity (*I*^2^ = 99%, *p* < 0.01). The highest SIV uptake was reported in the United States (76%), followed by Argentina (73%), while the lowest uptake was observed in Thailand (9%).

To determine if the study year, area, and sample size could contribute to the observed heterogeneity among studies, meta-regression and subgroup analyses were conducted. Univariate meta-regression revealed that there was no significant association between SIV uptake in the last flu season and study year, region, and sample size. In subgroup analyses, since all heterogeneity analyses were significant (*p* < 0.01), no variability could be explained (Table 3 and Supplementary Figure S1A–C).

**Figure 3:**
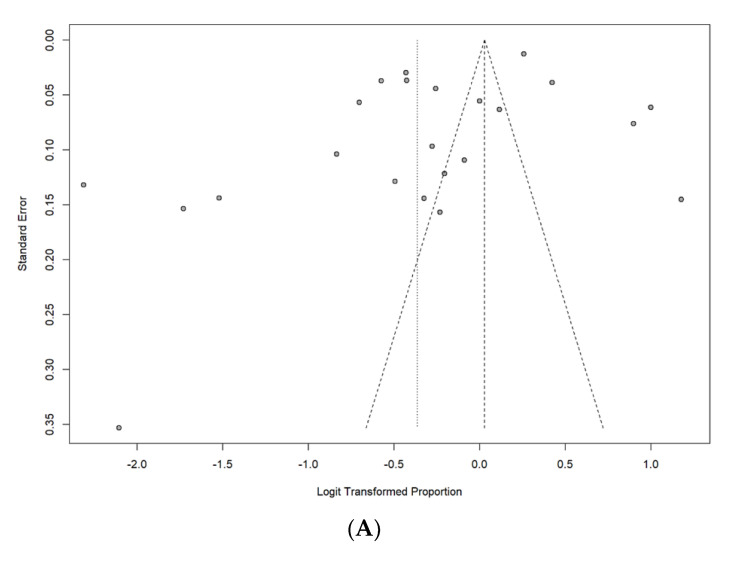

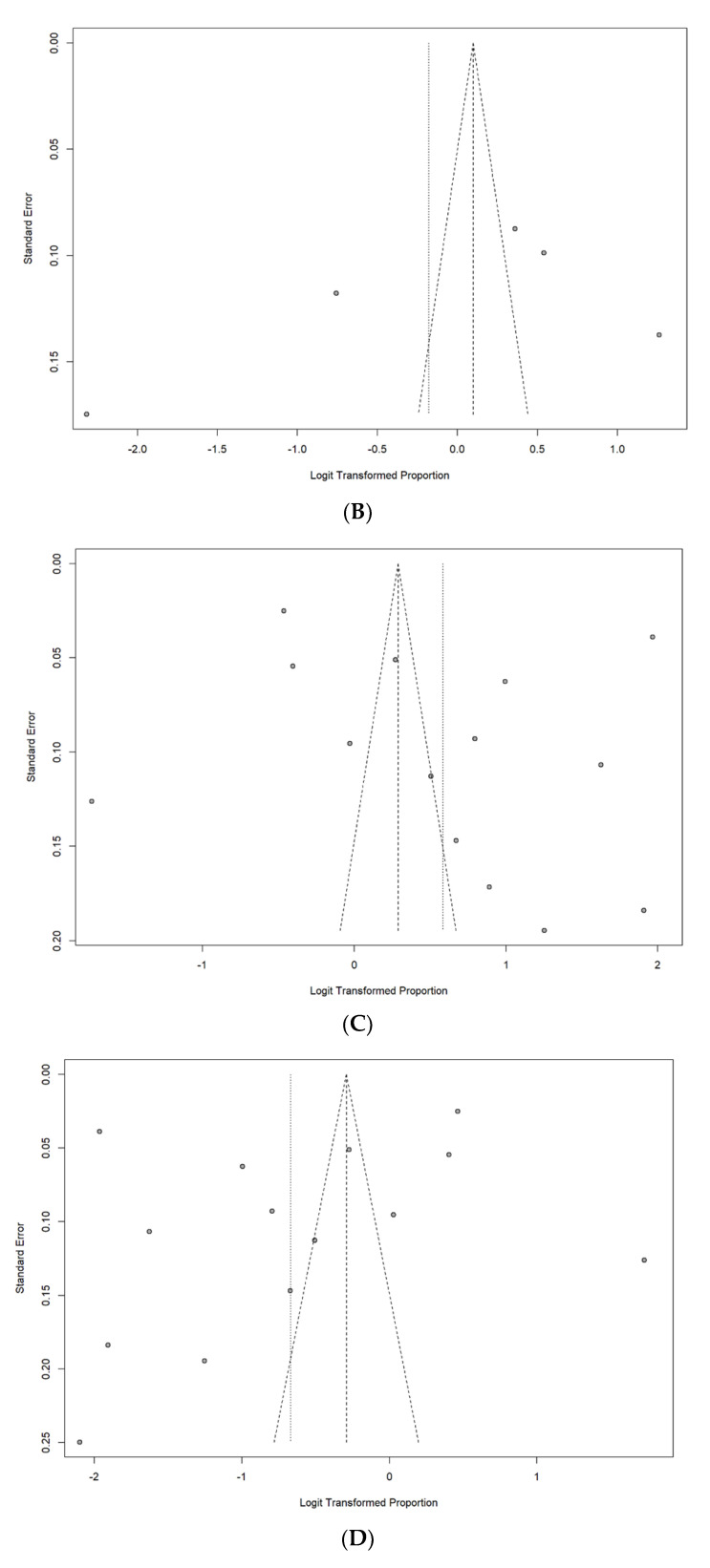
(**A**) Funnel plot of uptake rate of SIV in the last flu season; (**B**) funnel plot of uptake rate in lifetime; (**C**) funnel plot of acceptance rate of SIV; (**D**) funnel plot of hesitancy rate of SIV.

Publication bias among studies for SIV uptake in the last flu season was assessed using the funnel plot and Egger’s test. The funnel plot revealed that there was a probable publication bias toward studies with higher uptake rates using visual inspection (Figure 3A). However, Egger’s test showed no significant publication bias (*p* = 0.05). The adjusted uptake rate was 51% (95% CI: 41–62%) after filling in six missing studies by performing the trim-and-fill analysis.

#### 3.3.2. SIV Uptake in Lifetime among Children Aged 6–59 Months

Five studies reported on SIV uptake in the lifetime among children (Figure 2B). The overall uptake rate was 46% (95% CI: 20–74%), ranging from 9% to 78%, and with significant heterogeneity (*I*^2^ = 99%, *p* < 0.01). Poland reported the highest uptake of SIV in a lifetime (78%), whilst the lowest was in Hong Kong (9%).

Univariate meta-regression revealed that there was no significant association between SIV uptake in the lifetime and the study year, region, and sample size. In subgroup analyses, however, only the study year had no significant heterogeneity, which means that only the study year explained some of the variability among studies (Table 3 and Supplementary Figure S2A–C).

Concerning publication bias, the funnel plot showed a probable publication bias toward studies with higher uptake rates using visual inspection (Figure 3B). However, Egger’s test found no significant publication bias (*p* = 0.34). Moreover, no adjusted uptake rate could be generated after performing the trim-and-fill analysis.

#### 3.3.3. Parental Acceptance and Hesitancy of SIV

A total of fourteen studies were reported on parental acceptance and parental hesitancy (Figure 2C,D). The pooled prevalence of parental acceptance was 64% (95% CI: 51–75%), with a range of 15% to 88% (Figure 2C). Significant heterogeneity was found (*I*^2^ = 100%, *p* < 0.01). Canada reported the highest parental acceptance (88%), followed by the United States (87%) and South Korea (84%), whilst the Netherlands reported the lowest parental acceptance (15%). The pooled prevalence of parental hesitancy was 34% (95% CI: 22–48%), with a range of 11% to 85% (Figure 2D). Significant heterogeneity was also found (*I*^2^ = 100%, *p* < 0.01). The Netherlands reported the highest parental hesitancy (85%), while the lowest was found in Canada (12%) and the United States (11%).

Univariate meta-regression revealed that there was a significant association between parental acceptance and hesitancy and study region (*p* = 0.04, *p* = 0.02). The Americas had a higher parental acceptance than Asia (79% vs. 60%) and lower parental hesitancy than Asia (17% vs. 40%). In subgroup analyses, since all heterogeneity analyses were significant (*p* < 0.01), no variability could be explained. (Table 3 and Supplementary Figures S3A–C and S4A–C).

The funnel plot revealed a probable publication bias toward studies with low acceptance rates and high hesitancy rates (Figure 3C,D). However, Egger’s test showed no significant publication bias in parental acceptance and parental hesitancy (*p* = 0.39 and 0.35). Nevertheless, the adjusted acceptance and hesitancy rates were 46% (95% CI: 30–62%) and 54% (95% CI: 37–71%) after filling in six missing studies in acceptance and hesitancy using the trim-and-fill analyses.

#### 3.3.4. Factors Associated with Parental Acceptance/Hesitancy of SIV, and SIV Uptake in Children Aged 6–59 Months

Thirty-one studies reported on the factors associated with SIV (uptake in the last flu season, uptake in lifetime, acceptance/hesitancy), as shown in Table 2. Parental education level was a strong predictor reported in nine studies, with one study reporting a negative association [44] and some reporting a positive association with acceptance and uptake [19,20,37,38,40,45,50]. Household income [14,36,37,38,40,41,44,50] and having chronic diseases/comorbidities [35,36,41,47,48,51,52] were also important predictors of vaccine acceptance and uptake. The age of children had a positive association with vaccine acceptance/uptake [41,50] but a negative association in others [44,45,51,52]. Parental age was also a significant predictor of vaccine acceptance/hesitancy/uptake [19,25,35,36,38,44,45,50]. The cost of the vaccine had a negative association with acceptance and uptake [16,20,38,44,50,53]. Perceived susceptibility [35,41,45,48], perceived severity [41,43], perceived benefits [14,19,25,35,36,37,40,41,44,45,48,51], self-efficacy to receive vaccination [36,41], and cues to action [14,16,20,21,35,36,40,42,44,45,46,47,48] were also strong positive predictors of parental acceptance and the uptake of SIV among children. Nonetheless, perceived barriers such as serious side effects and safety concerns about the vaccines and inconvenience of vaccination centres were associated with higher parental hesitancy [28,35,40,45,48]. Other reported factors included ethnicity [51,54], employment status [25,35,38], the belief that SIV is the social norm [17,28,45], and awareness that children aged 5 years are the priority group [16,44,46].

#### 3.3.5. Parental Attitudes and Perceptions of SIV

A total of 17 studies reported parental attitudes and perceptions of SIV (Table 1). One revealed that the majority of the parents (about 90%) were aware of SIV [46], but another qualitative study showed that parents lacked knowledge about SIV [49]. The cost of the vaccine was an important issue mentioned in several studies [17,23,24,37,43]. SIV was perceived as safe, effective, and important [14,17,18,23,24,25,28,37,43,45,49]. However, worries about side effects and doubts about vaccine effectiveness were also reported [17,18,23,24,25,28,33,37,45,49]. The convenience of taking up SIV was also frequently discussed, with parents in some studies reporting that it was easy to take up SIV [17,24,28,45], but inconvenience was also reported in a few studies [37,49]. The belief that SIV was the social norm (e.g., many parents arranged SIV for their children) was revealed in various studies [18,23,24,25,49]. Nonetheless, worrying that their children could catch the flu from the vaccine was mentioned by parents [26,27,28,43]. Studies showed that parents were aware that the government recommended SIV for children aged less than 5 years or that physicians gave them the recommendations [14,23,24,25,27,33,37,45,49]. On the other hand, parents also thought that it was too young for their child to receive the SIV, and the vaccine would have a negative effect in the interactions with other vaccines to be received by the child [23,24,25]. Parents also had a high perceived self-efficacy in receiving SIV, as they believed that they would be able to get their child vaccinated if they desire to do so [23,24,25].

## 4. Discussion

To our knowledge, this is the first systematic review and meta-analysis to summarise evidence on SIV among children aged 6–59 months. This study revealed the pooled prevalence of parental acceptance, parental hesitancy, and the uptake of SIV in the last flu season and lifetime among children aged 6–59 months. Then, it assessed factors associated with SIV and parental attitudes and perceptions of SIV. Moreover, a reproducible search strategy with a well-established keyword system was employed for the identification of studies from key databases. All these are contributions to the literature.

This review found that the uptake of SIV in the last flu season was 41% (95% CI: 33–50%), SIV uptake in a lifetime was 46% (95% CI: 20–74%), parental acceptance was 64% (95% CI: 51–75%), and parental hesitancy was 34% (95% CI: 22–48%). Associated factors of the uptake, acceptance, or hesitancy included the age of the children or parents, parental education level, household income level, ethnicity, and other modifiable factors, including perceived benefits, perceived barriers, perceived severity, perceived susceptibility, and cues to action related to SIV.

Although 64% of the parents were willing to vaccinate their children, only 41% received SIV in the last flu season. This uptake rate is much lower than the target influenza vaccine coverage rate (VCR) of 75% recommended by the WHO [13]. This uptake is also slightly lower than that of their older counterparts (45.1–52.8%) [54,55]. This shows that there is a large room for improvement in the uptake of SIV in young children. Specific barriers to parents’ acceptance of SIV in their young children included their concerns that it was too young for their children to receive SIV and that SIV would have a negative effect in interaction with other vaccines to be received by the child [23,24,25]. Future SIV promotion programs should address these specific barriers in order to reduce parents’ concerns. For instance, supporting evidence emphasizing the safety of SIV in young children should be included in the promotion programs.

Additionally, meta-regression results showed that parental acceptance/hesitancy varied in different regions. The Americas had a higher acceptance than Asia (79% vs. 60%) and lower hesitancy than Asia (17% vs. 40%). The observed differences could be explained by the factors at the individual level and policy level. At the individual level, this study showed that a higher number of studies conducted in Asia (*n* = 6) than Americas (*n* = 3) reported perceived side effects of SIV as an important factor for acceptance of SIV. Additionally, the parental belief that it is too young for their child to receive SIV and SIV would have a negative effect in interaction with other vaccines to be received by the child were only reported in studies conducted in Asia [23,24,25]. Furthermore, these findings may be explained by the different vaccination policies in various countries. In the United States and Canada, SIV is included in the subsidised childhood immunisation scheme and free for children aged 6–59 months [56,57]. However, in countries in Asia, such as China and Singapore, SIV is not part of the subsidised childhood immunisation scheme, and it needs to be paid for out of pocket [40,46]. Health authorities in these Asian countries should consider providing subsidies for children aged 6–59 months to receive SIV.

Associated factors of the uptake/acceptance/hesitancy of SIV could be mainly categorised into individual and interpersonal levels based on the socioecological model [58]. Factors at the individual level included sociodemographics, such as the age of the children or parents, parental education level, household income level, ethnicity, and others. Older parents, lower parental education level, and household income were more consistently found to be negatively associated with uptake and acceptance. Therefore, in order to achieve the VCR (75%) recommended by the WHO [13], for successful vaccination programs in the future, such sociodemographic factors should be considered and incorporated when designing health promotion programs or targeted interventions. Other factors at the individual level also included some modifiable factors, such as perceived benefits, perceived barriers, perceived severity, and perceived susceptibility. Previous studies showed that interventions targeting modifiable factors (e.g., perceived benefits and perceived barriers) are effective in increasing vaccination uptake [59,60,61]. Factors at the interpersonal level included the influences of others (e.g., relatives/friends/spouses/health professionals). The study results consistently found that support from significant others and recommendations from health professionals to vaccinate their children had positive influences on parental decisions. Therefore, involving significant others and health professionals should be one of the core components in future health promotion programs or interventions for SIV among children.

This study also attempted to compare the uptake/acceptance/hesitancy of SIV before and during the coronavirus disease 2019 (COVID-19) pandemic. The interruptions in vaccination service provision (e.g., COVID-19 control measures and changes in the priority of health services) might play a role in the changes in the uptake/acceptance/hesitancy of SIV among children. Nonetheless, it did not show significant differences in the uptake/acceptance/hesitancy of SIV in this study. However, such results should be interpreted cautiously because there was a limited number of studies (*n* = 3) that were conducted during the COVID-19 pandemic, and thus, the impact of COVID-19 on children’s SIV was unclear. On the other hand, individual studies including older children (>59 months old) consistently reported a significant increase in parental acceptance (1.48–3.43-fold higher) after the COVID-19 outbreak [62,63,64]. For instance, one Chinese study found that parents were more willing to vaccinate their children against seasonal influenza than the time before the COVID-19 pandemic (68.4% vs. 35.2%) [62]. In Switzerland, compared to the time before the COVID-19 pandemic, about a two-fold increase in parental acceptance of SIV for their children was observed during the pandemic (17% vs. 7.2%) [63]. A similar trend was also reported among parents in Saudi Arabia [64]. Nevertheless, the above studies included children aged older than 59 months. Whether there is a change in the uptake/acceptance of SIV for children aged 6–59 months is still unclear.

Some studies suggested that certain strategies or interventions could increase SIV among children, including those conducted after the COVID-19 outbreak. One study showed that increasing the frequency of recommendations to receive SIV by health professionals could significantly increase SIV uptake among children [65]. Parental acceptance of SIV could be increased by offering free vaccination [62]. A review study revealed that vaccination reminders and education directed at either parents or healthcare providers, as well as via vaccination-related clinic process changes significantly improved SIV by 60% among children [66]. Several studies were conducted during the COVID-19 pandemic attempting to maintain or enhance children’s vaccination. These studies suggested using innovative approaches may be useful strategies for the post-pandemic period, such as using utilizing online technologies to remind parents and update them parents about their children’s vaccination status and remind them about the vaccination schedule [67,68,69], providing phone counselling interventions for children’s vaccination in parents [70], using telemedicine to provide necessary health information to assist parents in achieving self-efficacy and making decisions for their children’s vaccination [67,68,69,70,71,72,73], and using a chatbot real-time consultation messenger service to increase in parents’ motivation and self-efficacy to vaccinate their children [73].

Although this study provides valuable insights into parental acceptance/hesitancy and SIV uptake in the last flu season and lifetime among children aged 6–59 months, it has several limitations. First, high heterogeneity existed regarding the pooled prevalence of parental acceptance/hesitancy and uptake of SIV among children aged 6–59 months. The high heterogeneity could not be fully addressed despite subgroup analysis being performed. Nonetheless, these studies were from different countries/regions with different vaccination policies, and various types of study methodologies were used, which made heterogeneity inevitable. Second, data collected in the included studies in this review were self-reported, and recall and social desirability bias might exist. Third, since all studies were cross-sectional, no causal relationship between uptake/acceptance/hesitancy with factors could be established. Fourth, the exclusion of government documents/reports and newspapers without peer review might be justifiable for scientific rigour, but it is acknowledged that this decision might limit the inclusion of valuable information.

## 5. Conclusions

This study revealed the pooled prevalence of parental acceptance, parental hesitancy, and the uptake of SIV in the last flu season and lifetime, as well as their associated factors among children aged 6–59 months. The results revealed that the uptake of SIV in the last flu season, SIV uptake in a lifetime, parental acceptance, and parental hesitancy, were 41%, 46%, 64%, and 34%, respectively. Associated factors of the uptake, acceptance, or hesitancy included the age of the children or parents, parental education level, household income level, ethnicity, and other modifiable factors, including perceived benefits, perceived barriers, perceived severity, perceived susceptibility, and cues to action related to SIV. In order to achieve the VCR (75%) recommended by the WHO to protect children from vaccine-preventable diseases, it is crucial to implement tailored SIV interventions or programs for parents that address the factors, parental attitudes/perceptions, and specific local contexts in various regions or countries.

## Figures and Tables

**Table 1:** Characteristics of included studies.

Author	Study Year	Country/ Region	Study Design	Sample Size	Age of the Children	Uptake in the Last Flu Season/Uptake in Lifetime/Acceptance/Hesitancy	Key Findings
Bauer et al., 2021 [26]	2018	USA	Quantitative: cross-sectional survey	165	6–24 months	Uptake in the last flu season: 44% Acceptance: 71% Hesitancy: 11%	Uptake in the last flu season Barriers: forgetfulness, unawareness that the vaccine can be given at the age of 6 months, and beliefs that the vaccine is unnecessary and causes a flu-like reaction. Most helpful measures to reduce these barriers would be text message reminders, call reminders, and additional information or education on the influenza vaccine.
Biezen et al., 2018 [27]	2015	Australia	Qualitative: focus group	50	6–59 months	NA	Negative beliefs: the vaccine could cause influenza, and the vaccine was not necessary for their children. When SIV was recommended by their doctors and the influenza vaccine was part of the immunisation schedule, the parents would consider vaccinating their children.
Brown et al., 2011 [32]	2007	USA	Quantitative: cross-sectional survey	3072	6–59 months	Uptake in the last flu season: 39.5%	Uptake in the last flu season In total, 47.5% of the children received the influenza vaccine, and 39.5% had medical record verification.
Bults et al., 2011 [33]	2009	The Netherlands	Quantitative: cross-sectional survey	3127	6–59 months	Uptake in the last flu season: 36%	Uptake in the last flu season Reasons for taking up SIV: do not want their child to become sick, the government recommendation, and belief that they would regret if they do not do it. Reasons for refusing SIV: fear of side effects/harmful consequences, just having a bad feeling about it, and the vaccine was not thoroughly tested. More decliners than accepters experienced feelings of doubt about the vaccination decision, and decliners reported more often information-seeking behaviours. Decliners more frequently solicited advice from their social network than accepters. Accepters more often reported social influence on their vaccination decision and experienced more negative feelings after their vaccination decision.
Chantler et al., 2007 [34]	2005	UK	Mixed-method: Semi-structured interview and cross-sectional survey	Semi-structured interview: 10 Survey: 83	6–23 months	Uptake in the last flu season: 11%	Uptake in the last flu season Barriers: not convinced about the seriousness of paediatric influenza, difficult to differentiate influenza from other respiratory illnesses, expressed concerns about the need for annual injections and vaccine safety, and children should only be vaccinated if they are the main beneficiaries.
Chen et al., 2011 [35]	2009	Taiwan	Quantitative: cross-sectional survey	2778	6–36 months	Uptake in the last flu season: 60.4%	Uptake in the last flu season Associated factors: parental age, current employment, residence, children’s chronic diseases, hospitalisation, and influenza histories. Other associated factors: perceived susceptibility, perceived benefits, perceived barriers of vaccination, and cues to action.
Chen et al., 2015 [36]	2011	Taiwan	Quantitative: cross-sectional survey	1300	6–36 months	Uptake in the last flu season: 50% Acceptance: 73% Hesitancy: 27%	Acceptance Facilitators: perceived benefits, cues to action, and self-efficacy of vaccination against influenza, and children’s experience of influenza vaccinations in the last flu season.
Choi et al., 2017 [37]	2015	Republic of Korea	Quantitative: cross-sectional survey	639	<60 months	Acceptance: 83.6% Hesitancy: 16.4%	Acceptance: Facilitators: higher income and education and physicians’ recommendation. Barriers: cost of vaccination. Positive beliefs: influenza vaccine can reduce the symptoms and is effective at preventing the disease and safe for children. Negative beliefs: side effects of influenza vaccination, not convenient to receive the vaccination, and vaccination is not free.
Daley et al., 2007 [28]	2004	USA	Quantitative: cross-sectional survey	839	6–21 months	Uptake in the last flu season: 71%	Uptake in the last flu season Associated factors: the belief that SIV was the social norm, anticipating immunisation barriers. Parents hold a number of misperceptions about influenza vaccination, such as influenza vaccination could cause influenza infection (70%) and was not safe (21%).
Fisher et al., 2022 [14]	2016	Canada	Quantitative: cross-sectional survey	207	6–23 months	Acceptance: 66% Hesitancy: 34%	Acceptance Most parents considered immunisation to be safe, effective, and important; most perceived support for vaccination from significant others and clinicians. Associated factors: higher income, perceived vaccine safety, perceived benefit, perceived cues to action, and perceived support from significant others.
Frawley et al., 2020 [16]	2018	Australia	Quantitative: cross-sectional survey	1002	6–59 months	Uptake in the last flu season: 52.9%	Uptake in the last flu season Associated factors: being a mother, not having a healthcare card, the vaccine was free, and recommended by a pharmacist Negative beliefs: safety concerns of the influenza vaccine, and their child can catch influenza from the vaccine.
Ganczak et al., 2013 [38]	2010	Poland	Quantitative: cross-sectional survey	308	6–59 months	Uptake in lifetime: 77.9%	Uptake in lifetime Parental decision was based on a healthcare worker’s recommendation. Associated factors: parental age, high socioeconomic status, having one child, convenience of vaccination, and the vaccine was not free.
Gentile et al., 2015 [39]	2013	Argentina	Quantitative: cross-sectional survey	1350	6–24 months	Uptake in the last flu season: 73.1%	Uptake in the last flu season Factors associated with a decreased risk of delayed schedules were perception of the importance of influenza vaccination, having less than one year of age, and having received information in paediatric visits or in any media.
Han et al., 2022 [40]	2021	China	Quantitative: cross-sectional survey	2081	<5 years	Uptake in the last flu season: 43.63%	Uptake in the last flu season Facilitators included caregivers who had a bachelor’s degree or above, an annual household income of RMB <20,000, confidence in the importance, safety, and effectiveness of influenza vaccine, knowing other caregivers were vaccinating their children, and receiving positive influence from healthcare workers and family members. Barriers included poor access, including conflicts between caregivers’ availability and vaccination service schedules and inconvenient transportation to the vaccination site.
He et al., 2015 [41]	2013	China	Quantitative: cross-sectional survey	335	6–36 months	Uptake in the last flu season: 47.7% Acceptance: 62.4% Hesitancy: 37.6%	Uptake in the last flu season Associated factors: household income, children’s age, social norm and perceived control, and perceived safety. Associated factors: household income, children with a history of taking seasonal influenza vaccine, perceived children’s health status, worry/anxiety about their children’s influenza infection, perceived control, perceived safety, perceived benefits, social norm, and perceived severity.
Hemingway et al., 2004 [42]	2003	USA	Quantitative: cross-sectional survey	329	6–59 months	Uptake in the last flu season: 18%	Uptake in the last flu season Associate factors: parental recall of a physician recommendation and family member vaccinated. In total, 65% of parents of high-risk children did not recall a physician’s recommendation, 62% learned of influenza vaccination from the mass media, and 38% from their paediatricians
Humiston et al., 2005 [43]	2004	USA	Quantitative: cross-sectional survey	153	6–23 months	Acceptance: 78% Hesitancy: 22%	Acceptance Negative beliefs: the vaccine was not safe, and the child could catch influenza from vaccination. Associated factors: perceived severity of influenza, perceived safety of the vaccine, and parental educational level.
Lai et al., 2022 [44]	2019	China	Quantitative: cross-sectional survey	6668	6–59 months	Acceptance: 38.6% Hesitancy: 61.4%	Hesitancy Associated factors: perceiving high importance, safety or efficacy) of SIV, knowing children as a priority group, and trusting vaccination advice from medical staff. Others included: sociodemographic characteristics, such as children’s age, the number of children in the family and caregivers’ information (age, family relation, education level, basic medical insurance type, religious beliefs, income, place of residence, and province).
Lau et al., 2021 [45]	2011	Hong Kong	Quantitative: cross-sectional survey	540	24–59 months	Acceptance: 68.9% Hesitancy: 31.1%	Acceptance Being a mother, having a child aged ≥48 months, and the absence of IV experience in family members were the significant background factors of parental intention in the ever-vaccinated group, while being a mother, younger, and more educated were significant factors of parental intention in the never-vaccinated group. Perceived susceptibility, perceived benefit, perceived barrier, cue to action, subjective norm, and having family members vaccinated were associated with parental intention for ever-vaccinated children’s IV, while only perceived benefit and subjective norm were significant for never-vaccinated children.
Lau et al., 2013 [23]	2006	Hong Kong	Quantitative: cross-sectional survey	401	6–23 months	Uptake in lifetime: 9%	Uptake in lifetime Associated factors: physician recommendations and perceived side effects of SIV. Belief that SIV could reduce the risk of influenza-induced complications (70.5%), hospitalisation (70.5%), and death (65.9%).
Low et al., 2017 [46]	2016	Singapore	Quantitative: cross-sectional survey	332	6 months–5 years	Uptake in the last flu season: 15% Uptake in lifetime: 32%	Uptake in the last flu season Associated factors: being recommended influenza vaccine by a child’s doctor, receiving influenza vaccine information from a private general practitioner, regularly receiving pre-travel influenza vaccine, parental willingness, being well informed about SIV knowledgeable about influenza with a score of 12 out of 15) and influenza vaccine with 88% of respondents were aware that there was a vaccine for influenza.
Ma et al., 2006 [47]	2004	USA	Quantitative: cross-sectional survey	256	6–59 months	Uptake in the last flu season: 38%	Uptake in the last flu season Associated factors: recalling a physician’s recommendation, having a family member who had received the influenza vaccine, having a continuity clinic visit, and having a high-risk medical condition.
Nowalk et al., 2005 [48]	2003	USA	Quantitative: cross-sectional survey	436	6–23 months	Uptake in the last flu season: 43.2%	Uptake in the last flu season Associated factors: perceived doctor’s recommendation, relatives and friends thinking that child should have a flu shot, the belief that a child with asthma should receive a flu shot, the belief that receiving an influenza shot is a smart idea, the belief that unvaccinated child will get the flu, and belief that flu shot prevents flu, worrying that child will get the flu from the flu shot, and the belief that the flu shot is more trouble than it is worth.
Nowalk et al., 2007 [21]	2004	USA	Quantitative: cross-sectional survey	274	6–23 months.	Uptake in the last flu season: 45%	Uptake in the last flu season Associated factors: doctor’s recommendation, receiving a reminder and belief that their young child should be vaccinated.
Price et al.,, 2022 [49]	2020	UK	Qualitative: semi-structured interview	12	2–3 years	NA	Facilitators included knowledge and awareness of IV, trust in the vaccine, access to vaccine information, physician recommendation, access to vaccination, perceived severity, social norms, the belief that vaccinating a child protects the wider family, and the belief that vaccinating a child is a social responsibility. Barriers included a lack of knowledge about the flu, the belief that one’s child is not at risk from the flu, and the belief that catching the flu is inevitable for children, inconvenient appointments, lack of access due to a vaccine shortage, and lack of awareness about their child’s eligibility, and the fear of side-effects and frustration with or distrust of medical professionals.
Schuller et al., 2013 [50]	2008	USA	Quantitative: cross-sectional survey	25,256	19–35 months	Uptake in the last flu season: 56.4%	Uptake in the last flu season Associated factors: primary caregiver being older, married, and more educated, a child with private insurance, younger children and first-born children, Black children were the least likely to have been immunised, an income above USD 75,000 per year, a small household size, and residence in the northeast were associated with an increased probability of immunisation, whereas living in the south was associated with decreased likelihood.
Schellenberg et al., 2023 [51]	2018	Canada	Quantitative: cross-sectional survey	6125	24 months	Acceptance: 87.7% Hesitancy: 12.3%	Hesitancy Female parents, the child had health issues or was too young, poor knowledge, and attitudes and beliefs increased likelihood of refusal.
Shinall et al., 2007 [52]	2005	USA	Quantitative: cross-sectional survey	198	6–59 months	Uptake in the last flu season: 42%	Uptake in the last flu season More children who were 6 to 23 months than those who were 24 to 59 months of age were vaccinated. Children with chronic medical conditions were more likely to be vaccinated than healthy children who were 24 to 59 months of age.
Thanee et al., 2021 [20]	2019	Thailand	Quantitative: cross-sectional survey	700	6–36 months	Uptake in the last flu season: 9%	Uptake in the last flu season Caregivers of the vaccinated children were more likely to have higher education and to know of influenza illness. Factors associated with children receiving influenza vaccination were identifying healthcare providers as a primary source of information about influenza illness for parents, parents’ strongly agreeing with the national recommendation for influenza vaccination in young children, using health insurance provided by the government or parent’s employer for children’s doctor visits, and the children’s history of receiving influenza vaccination in the 2017 season or earlier.
van Lier et al., 2017 [18]	2012	The Netherlands	Quantitative: cross-sectional survey	491	0–48 months	Acceptance: 15% Hesitancy: 85%	Acceptance Main drivers of intention were the perceived importance of vaccination against the particular disease and the perception of whether or not the disease is severe enough to justify vaccination.
Wei et al., 2021 [17]		China	Quantitative: cross-sectional survey	1564	0–59 months	Acceptance: 56.8% Hesitancy: 43.2%	Hesitancy From rural areas, do not know the government recommendation for flu vaccination, do not know the flu vaccine is vaccinated annually, and family members, friends, and neighbours had a positive attitude toward flu vaccine were related factors of the guardians’ IVH
Williams et al., 2021 [53]	2020	USA	Quantitative: cross-sectional survey	263	2 years	Uptake in the last flu season: 76.5% Acceptance: 87% Hesitancy: 13%	Hesitancy Caccine-hesitant parents were more likely to be English-speaking, non-Latino race/ethnicity, non-publicly insured, and born in the United States.
Wu et al., 2015 [24]	2011	Hong Kong	Quantitative: cross-sectional survey	540	24–59 months	Uptake in lifetime: 58.9%	Uptake in lifetime Associated factors: family members’ SIV experience, perceived susceptibility to seasonal influenza, perceived benefits of IV, perceived barriers of IV, cue to action, and supportive subjective norm.
Wu et al., 2020 [25]	2011	Hong Kong	Quantitative: cross-sectional survey	440	24–59 months	Uptake in the last flu season: 30.22% Uptake in lifetime: 63.2% Acceptance: 49.3% Hesitancy: 50.7%	Uptake in the last flu season Facilitators included caregivers being female, parental positive attitude, norm, and behavioural intention. Uptake in lifetime Facilitators included positive attitudes (e.g., belief that IV is effective) and perceived norms, whilst barriers included negative attitudes. Acceptance Facilitators included visiting doctor before, positive attitudes, and perceived norms, whilst barriers included part-time/unemployed,
Wu et al., 2023 [19]	2019	China	Quantitative: cross-sectional survey	1404	0–59 months	Uptake in the last flu season: 33.1% Hesitancy: 40% Hesitancy: 60%	Uptake in the last flu season Associated factors included guardians’ gender being male, older age, lower education level, perceived benefit, knowing that government recommends influenza vaccination, that the influenza vaccine is recommended to be taken annually, those who reported that their communities promote influenza vaccination, with support of their family members, friends and neighbours, and health care workers, and hesitancy. Hesitancy Associated factors included relative knowledge and social influence.
Zhang et al., 2022 [15]	2018	China	Quantitative: cross-sectional survey	4719	6–59 months	Uptake in the last flu season: 39.4%	Uptake in the last flu season Associated factors: older child age, living in Southern China were more likely to vaccinate.

**Table 2:** Summary of the uptake of seasonal influenza vaccination in the last flu season, uptake in lifetime, parental acceptance, and parental hesitancy from the included studies.

Author, Year	Country/Region	Sample Size	Uptake in the Last Flu Season	Uptake in Lifetime	Acceptance	Hesitancy	Associated Factors
Bauer et al., 2021 [26]	USA	165	44%	-	71%	11%	-
Brown et al., 2011 [32]	USA	3072	39.5%	-	-	-	-
Bults et al., 2011 [33]	The Netherlands	3127	36%	-	-	-	Uptake in the last flu season: experienced feelings of doubt about the vaccination decision (−); information-seeking behaviour (−); social influence on their vaccination decision (+)
Chantler et al., 2007 [34]	UK	83	11%	-	-	-	-
Chen et al., 2011 [35]	Taiwan	2778	60.4%	-	-	-	Uptake in the last flu season: age of the caregiver (−); unemployed (+); living in rural areas (+); chronic disease (−); hospitalisation history (−); influenza histories of the child (+); perceived susceptibility (+)/perceived benefits of vaccinations (+); perceived barriers (−); cues to action (+)
Chen et al., 2015 [36]	Taiwan	1300	50%	-	73%	27%	Uptake in the last flu season: age of the caregiver (+); female gender (−); average household income (−); being mother compared to father(−); being grandfather compared to father (+); influenza vaccination of the children in the last year (+); children’s average frequency of cold per year (+); perceived benefits of the vaccination (+); cues to action (+); self-efficacy of childhood vaccination (+)
Choi et al., 2017 [37]	Republic of Korea	639	-	-	83.6%	16.4%	Uptake in the last flu season: household income (+); parental education level (+)
Daley et al., 2007 [28]	USA	839	71%	-	-	-	Uptake in the past year: belief that IV is the social norm (+); perceived barriers (−)
Fisher et al., 2022 [14]	Canada	207	-	-	66%	34%	Acceptance: household income (+); perceived vaccine safety (+); perceived benefit (+); cues to action (+); perceived support from significant others (+)
Frawley et al., 2020 [16]	Australia	1002	52.9%	-	-	-	Uptake in the last flu season: being mother (−); having a health care card (+); know the influenza vaccine was free for the child (+); a recommendation from a health professional (+)
Ganczak et al., 2013 [38]	Poland	308	-	77.9%	-	-	Uptake in lifetime: age of the parent (+); socio-economic status (+); having only one child (+); health system factor, i.e., practice location (+); cost of a vaccine (−)
Gentile et al., 2015 [39]	Argentina	1350	73.1%	-	-	-	Uptake in the last flu season: perceived importance of IV (+)
Han et al., 2022 [40]	China	2081	43.6%	-	-	-	Uptake in the last flu season: parental education level (+); household income (−); confidence in the importance, safety, and effectiveness of influenza vaccine (+); knowing other caregivers were vaccinating their children (+); receiving positive influence from healthcare workers and family members (+); poor access, including conflicts between caregivers’ availability and vaccination service schedules and inconvenient transportation to the vaccination site (−)
He et al., 2015 [41]	China	335	47.7%	-	62.4%	37.6%	Uptake in the last flu season: household income (+); age of the child (+); social norm (+); perceived control (+); perceived vaccine safety (+) Acceptance: household income (+); children with a history of taking SIV (+); perceived children’s health status (+); worry/anxious about their children influenza infection (+); perceived control (+); perceived vaccine safety (+); perceived benefit (+); social norm (+); perceived severity (+)
Hemingway et al., 2004 [42]	USA	329	18%	-	-	-	Uptake in the last flu season: a physician’s recommendation (+); family members vaccinated (+)
Humiston et al., 2005 [43]	USA	153	-	-	78%	22%	Acceptance: perceived severity (+); perceived vaccine safety (+); perceived importance to vaccinate the child (+); parental education level (+)
Lai et al., 2022 [44]	China	6668	-	-	38.6%	61.4%	Hesitancy: perceiving importance to vaccinate the child (−); perceived vaccine safety (−); perceived vaccine efficacy (−); know children as a priority group (−); trusting vaccination advice from medical staff (−); perceived barriers (+); age of the child (−); the number of children in the family (−); caregivers’ age (−); parental education level (−); household income (+)
Lau et al., 2021 [45]	Hong Kong	540	-	-	68.9%	31.1%	Acceptance: being a mother (−); age of the child (−); positive influenza vaccination experience in family members (+); age of the parents (−); parental education level (+); perceived susceptibility (+); perceived benefit (+); perceived barrier (−); cue to action (+); subjective norm (+); having family members vaccinated (+)
Lau et al., 2013 [23]	Hong Kong	401	-	9%	-	-	Uptake in lifetime: a physician’s recommendations (+); parental perceptions of the side effects of IV (−)
Low et al., 2017 [46]	Singapore	332	15%	32%	-	-	Uptake in the last flu season: a physician’s recommendation (+); the child was vaccinated before (+); willingness to vaccinate (+); feeling well-informed about influenza vaccine (+); receiving influenza vaccine information from television (−)
Ma et al., 2006 [47]	USA	256	38%	-	-	-	Uptake in the last flu season: a physician’s recommendation (+); having a family member vaccinated (+); having a continuity clinic visit (+); having a high-risk medical condition (+)
Nowalk et al., 2005 [48]	USA	436	43.2%	-	-	-	Uptake in the last flu season: a physician’s recommendation (+); relatives and friends think that child should have flu shot (+); the belief that a child with asthma should receive a flu shot (+); the belief that receiving an influenza shot is a smart idea (+); the belief that unvaccinated child will get flu (+); belief that flu shot prevents flu (+); worrying that child will get the flu from flu shot (−); belief that the flu shot is more trouble than it is worth (−)
Nowalk et al., 2007 [21]	USA	274	45%	-	-	-	Uptake in the last flu season: a physician’s recommendation (+); receiving a reminder (+); the parental belief that the child should be vaccinated (+)
Schuller et al., 2013 [50]	USA	25,256	56.4%	-	-	-	Uptake in the last flu season: age of the parent (+); married (+); parental education level (+); a child with private insurance (+); age of the child (+); first-born child (+); ethnicity: black (+); household income level (+)
Schellenberg et al., 2023 [51]	Canada	6125	-	-	87.7%	12.3%	Hesitancy: female parents (−); child had health issues (−); age of the child (−); knowledge, attitudes and belief (−)
Shinall et al., 2007 [52]	USA	198	42%	-	-	-	Uptake in the last flu season: age of the child (−); child with chronic medical conditions (+)
Thanee et al., 2021 [20]	Thailand	700	9%	-	-	-	Uptake in the last flu season: parental education level (+); knowledge about influenza illness (+); healthcare providers as a primary source of information about influenza illness for parents (+); parents’ strongly agreeing with the national recommendation for influenza vaccination in young children (+); using health insurance provided by the government or parent’s employer for children’s doctor visits (+); the children’s history of receiving influenza vaccination (+)
van Lier et al., 2017 [18]	The Netherlands	491	-	-	15%	85%	Acceptance: perceived importance of vaccination against the particular disease (+); the perception of whether or not the disease is severe enough to justify vaccination (+)
Wei et al., 2021 [17]	China	1564	-	-	56.8%	43.2%	Hesitancy: living in a rural area (+); do not know the government recommendation for flu vaccination (+); do not know the flu vaccine is vaccinated annually (+); family members, friends and neighbours had a positive attitude toward flu vaccine (−)
Williams et al., 2021 [53]	USA	263	76.5%	-	87%	13%	Hesitancy: English-speaking (+); non-Latino race/ethnicity (+); non-publicly insured (+); born in the United States (+)
Wu et al., 2015 [24]	Hong Kong	540	-	58.9%	-	-	Uptake in lifetime: family members vaccinated (+); perceived susceptibility of seasonal influenza (+); perceived benefits of IV (+); perceived barriers of IV (−); cue to action (+); supportive subjective norm (+)
Wu et al., 2020 [25]	Hong Kong	440	30.2%	63.2%	49.3%	50.7%	Uptake in the last flu season: caregivers being female (+); parental positive attitude (+); subjective norm (+); behavioral intention (+) Uptake in a lifetime: positive attitudes (e.g., belief that IV is effective) (+); perceived norm (+); negative attitudes (−) Acceptance: visiting doctor before (+); positive attitudes (+); perceived norm (+); parttime/unemployed (−); child’s uptake in the past year (+)
Wu et al., 2023 [19]	China	1404	33.1%	-	40.0%	60.0%	Uptake in the last flu season: caregiver being male (+); the age of the parent (+); parental education level (+); perceived benefit (+); knowing that government recommends influenza vaccination (+); knowing that the influenza vaccine is recommended to be taken annually (+); those who reported that their communities promote influenza vaccination (+); with support of their family members, friends and neighbours, and HCWs (+); hesitancy (−) Hesitancy: relative knowledge (−); social influence (−)
Zhang et al., 2022 [15]	China	4719	39.4%	-	-	-	Uptake in the last flu season: age of the child (+); living in Southern China (+)

+ indicates positive association; − indicates negative association.

**Table 3:** Moderators of vaccine uptake, acceptance, and hesitancy rates of SIV (meta-regression and subgroup analyses).

Moderator	Number of Studies	Proportion of Outcome (95% CI)	Heterogeneity		Moderator Effect (Meta-Regression)	
					Univariate	
			*I*^2^ Within	*p*-Value	Coefficient	*p*-Value
**Uptake in the last flu season**						
**Study year**					0.01	0.87
2010 or before	11	0.41 (0.30–0.53)	99%	<0.01		
2011–2015	4	0.51 (0.33–0.68)	99%	<0.01		
2016–2019	6	0.29 (0.16–0.47)	99%	<0.01		
2020 or after	2	0.61 (0.28–0.96)	99%	<0.01		
**Region**					0.03	0.61
Asia	9	0.34 (0.23–0.48)	99%	<0.01		
Americas	11	0.50 (0.38–0.61)	99%	<0.01		
Europe	2	0.21 (0.06–0.55)	95%	<0.01		
Oceania	1	0.53 (0.50–0.56)	-	-		
**Sample size**					0.11	0.17
≤1000	13	0.35 (0.23–0.49)	99%	<0.01		
>1000	10	0.48 (0.40–0.57)	99%	<0.01		
**Uptake in lifetime**						
**Study year**					−0.02	0.91
2010 or before	2	0.37 (0.02–0.95)	100%	<0.01		
2011–2015	2	0.61 (0.57–0.65)	47%	0.17		
2016–2019	1	0.32 (0.27–0.37)	-	-		
**Region**					0.19	0.28
Asia	4	0.37 (0.14–0.68)	99%	<0.01		
Americas	0	-	-	-		
Europe	1	0.78 (0.73–0.82)	-	-		
Oceania	0	-	-	-		
**Sample size**					-	-
≤1000	5	0.46 (0.20–0.74)	99%	<0.01		
>1000	0	-	-	-		
**Parental acceptance**						
**Study year**					0.02	0.77
2010 or before	1	0.78 (0.70–0.84)	-	-		
2011–2015	6	0.59 (0.36–0.78)	99%	<0.01		
2016–2019	6	0.62 (0.44–0.77)	100%	<0.01		
2020 or after	1	0.87 (0.82–0.91)	-	-		
**Region**					0.19	0.04
Asia	8	0.60 (0.48–0.71)	99%	<0.01		
Americas	5	0.79 (0.70–0.87)	96%	<0.01		
Europe	1	0.15 (0.12–0.19)	-	-		
Oceania	0	-	-	-		
**Sample size**					−0.05	0.66
≤1000	9	0.66 (0.49–0.79)	99%	<0.01		
>1000	5	0.62 (0.40–0.80)	100%	<0.01		
**Parental hesitancy**						
**Study year**					−0.04	0.68
2010 or before	1	0.22 (0.16–0.30)	-	-		
2011–2015	6	0.41 (0.22–0.64)	93%	<0.01		
2016–2019	6	0.34 (0.17–0.55)	100%	<0.01		
2020 or after	1	0.13 (0.09–0.18)	-	-		
**Region**					0.22	0.02
Asia	8	0.40 (0.29–0.52)	99%	<0.01		
Americas	5	0.17 (0.11–0.26)	95%	<0.01		
Europe	1	0.85 (0.81–0.88)	-	-		
Oceania	0	-	-			
**Sample size**					0.07	0.57
≤1000	9	0.31 (0.18–0.50)	99%	<0.01		
>1000	5	0.38 (0.20–0.60)	100%	<0.01		

## Data Availability

Data supporting this systematic review are available in the reference section. In addition, the analysed data used in this systematic review are available from the author upon reasonable request.

## Supplementary Materials

The following supporting information can be downloaded at: https://www.mdpi.com/article/10.3390/vaccines11081360/s1. Table S1 (A): Quality assessment of quantitative studies; (B): Quality assessment of qualitative studies; (C): Quality assessment of mixed-method study; Figure S1 (A): Forest plot of subgroup analysis of the uptake in the last flu season by year; (B): Forest plot of subgroup analysis of the uptake in the last flu season by region; (C): Forest plot of subgroup analysis of the uptake in the last flu season by sample size; Figure S2 (A): Forest plot of subgroup analysis of the uptake in lifetime by year; (B): Forest plot of subgroup analysis of the uptake in lifetime by region; (C): Forest plot of subgroup analysis of the uptake in lifetime by sample size; Figure S3 (A): Forest plot of subgroup analysis of the parental acceptance by year; (B): Forest plot of subgroup analysis of the parental acceptance by region; (C): Forest plot of subgroup analysis of the parental acceptance by sample size; Figure S4 (A): Forest plot of subgroup analysis of the parental hesitancy by year; (B): Forest plot of subgroup analysis of the parental hesitancy by region; (C): Forest plot of subgroup analysis of the parental hesitancy by sample size
