# Supplementary material for: Parental Acceptance, Parental Hesitancy, and Uptake of Seasonal Influenza Vaccination among Children Aged 6–59 Months: A Systematic Review and Meta-Analysis
Source: Vaccines (Basel). 2023 Aug 13;11(8):1360. doi: 10.3390/vaccines11081360 (PMC10459009; doi:10.3390/vaccines11081360)
Supplement: Supplementary file 1 [file vaccines-11-01360-s001.zip › vaccines-2522495-supplementary.pdf]

Table S1A Quality assessment of quantitative studies

Author	Study design	Is the sampling strategy relevant to address the quantitative research question?	Is the sample representative of the population under study?	Are measurements appropriate (clear origin, or validity known, or standard instrument)?	Is there an acceptable response rate (60% or above)?
Bauer et al. 2021	Quantitative: cross-sectional survey	Yes	No	Yes	No
Brown et al. 2011	Quantitative: cross-sectional survey	Yes	Yes	Yes	Can't tell
Bults et al. 2011	Quantitative: cross-sectional survey	Yes	Yes	Yes	Yes
Chantler et al. 2007	Mixed-method: Semi-structured interview and cross-sectional survey	Yes	No	Yes	No
Chen et al. 2011	Quantitative: cross-sectional survey	Yes	Yes	Yes	Yes
Chen et al. 2015	Quantitative:	Yes	Yes	Yes	No

	cross-sectional survey				
Choi et al. 2017	Quantitative: cross-sectional survey	Yes	Yes	Yes	Can’t tell
Daley et al. 2007	Quantitative: cross-sectional survey	Yes	Yes	Yes	No
Fisher et al. 2022	Quantitative: cross-sectional survey	Yes	Yes	No	Can’t tell
Frawley et al. 2020	Quantitative: cross-sectional survey	Yes	Yes	Yes	No
Ganczak et al. 2013		Yes	Yes	Yes	Yes
Gentile et al. 2015	Quantitative: cross-sectional survey	Yes	Yes	Yes	Can’t tell
Han et al. 2022	Quantitative: cross-sectional survey	Yes	Yes	Yes	Can’t tell
He et al. 2015	Quantitative: cross-sectional survey	Yes	No	Yes	Can’t tell
Hemingway et al.	Quantitative:	Yes	No	Yes	Can’t tell

2004	cross-sectional survey				
Humiston et al. 2005	Quantitative: cross-sectional survey	Yes	No	Yes	Can’t tell
Lai et al. 2022	Quantitative: cross-sectional survey	Yes	Yes	Yes	Can’t tell
Lau et al. 2021	Quantitative: cross-sectional survey	Yes	Yes	Yes	Yes
Lau et al. 2013	Quantitative: cross-sectional survey	Yes	Yes	Yes	Yes
Low et al. 2017	Quantitative: cross-sectional survey	Yes	Yes	Yes	Can’t tell
Ma et al. 2006	Quantitative: cross-sectional survey	Yes	No	Yes	Yes
Nowalk et al. 2005	Quantitative: cross-sectional survey	Yes	Yes	Yes	No
Nowalk et al. 2007	Quantitative: cross-sectional	Yes	Yes	Yes	No

	survey				
Schuller et al. 2013	Quantitative: cross-sectional survey	Yes	Yes	Yes	Can’t tell
Schellenberg et al. 2023	Quantitative: cross-sectional survey	Yes	Yes	Yes	Yes
Shinall et al. 2007	Quantitative: cross-sectional survey	Yes	No	Yes	Yes
Thanee et al. 2021	Quantitative: cross-sectional survey	Yes	Yes	Yes	Can’t tell
van Lier et al. 2017	Quantitative: cross-sectional survey	Yes	Yes	Yes	No
Wei et al. 2021	Quantitative: cross-sectional survey	Yes	Yes	Yes	Yes
Williams et al. 2021	Quantitative: cross-sectional survey	Yes	Yes	Yes	Yes
Wu et al. 2015	Quantitative: cross-sectional survey	Yes	Yes	Yes	Can’t tell

Wu et al. 2020	Quantitative: cross-sectional survey	Yes	Yes	Yes	Yes
Wu et al. 2023	Quantitative: cross-sectional survey	Yes	Yes	Yes	Can’t tell
Zhang et al. 2022	Quantitative: cross-sectional survey	Yes	Yes	Yes	No

Table S1B Quality assessment of qualitative studies

Author	Study design	Are the sources of qualitative data (archives, documents, informants, observations) relevant to address the research question (objective)?	Is the process for analyzing qualitative data relevant to address the research question (objective)?	Is appropriate consideration given to how findings relate to the context, e.g., the setting, in which the data were collected?	Is appropriate consideration given to how findings relate to researchers' influence, e.g., through their interactions with participants?
Biezen et al. 2018	Qualitative: focus group	Yes	Yes	Yes	No
Chantler et al. 2007	Mixed-method: Semi-structured interview and cross-sectional survey	Yes	Yes	Yes	No
Price et al., 2022	Qualitative: semi-structured interview	Yes	Yes	Yes	No

Table S1C Quality assessment of mixed-method study

Author	Study design	Is the mixed methods research design relevant to address the qualitative and quantitative research questions (or objectives), or the qualitative and quantitative aspects of the mixed methods question (or objective)?	Is the integration of qualitative and quantitative data (or results) relevant to address the research question (objective)?	Is appropriate consideration given to the limitations associated with this integration, e.g. the divergence of qualitative and quantitative data?
Chantler et al. 2007	Mixed-method: Semi-structured interview and cross-sectional survey	Yes	Yes	No

**year2 = 3**			
Bauer, 2021	0.44	[0.37; 0.52]	4.7%
Frawley, 2020	0.53	[0.50; 0.56]	4.8%
Low, 2017	0.15	[0.11; 0.19]	4.7%
Thanee, 2021	0.09	[0.07; 0.11]	4.7%
Wu, 2023	0.33	[0.31; 0.36]	4.8%
Zhang, 2022	0.39	[0.38; 0.41]	4.8%
**Random effects model**	**0.29**	**[0.16; 0.47]**	**28.5%**
Heterogeneity: $I^2 = 99\%$, $\chi^2_5 = 368.34$ ($p < 0.01$)			
**year2 = 1**			
Bults, 2011	0.36	[0.34; 0.38]	4.8%
Chen, 2011	0.60	[0.59; 0.62]	4.8%
Daley, 2007	0.71	[0.68; 0.74]	4.8%
Hemingway, 2004	0.18	[0.14; 0.23]	4.7%
Ma, 2006	0.38	[0.32; 0.44]	4.7%
Nowalk, 2005	0.43	[0.38; 0.48]	4.8%
Nowalk, 2007	0.45	[0.39; 0.51]	4.7%
Schuller, 2013	0.56	[0.56; 0.57]	4.8%
Shinall, 2007	0.42	[0.35; 0.49]	4.7%
**Random effects model**	**0.45**	**[0.34; 0.56]**	**42.9%**
Heterogeneity: $I^2 = 99\%$, $\chi^2_8 = 787.84$ ($p < 0.01$)			
**year2 = 2**			
Chen, 2015	0.50	[0.47; 0.53]	4.8%
Gentile, 2015	0.73	[0.71; 0.75]	4.8%
He, 2015	0.48	[0.42; 0.53]	4.8%
Wu, 2020	0.30	[0.26; 0.35]	4.8%
**Random effects model**	**0.51**	**[0.33; 0.68]**	**19.1%**
Heterogeneity: $I^2 = 99\%$, $\chi^2_3 = 288.72$ ($p < 0.01$)			
**year2 = 4**			
Han, 2022	0.44	[0.41; 0.46]	4.8%
Williams, 2021	0.76	[0.71; 0.81]	4.7%
**Random effects model**	**0.61**	**[0.28; 0.86]**	**9.5%**
Heterogeneity: $I^2 = 99\%$, $\chi^2_1 = 88.96$ ($p < 0.01$)			
**Random effects model**	**0.43**	**[0.34; 0.52]**	**100.0%**
Heterogeneity: $I^2 = 99\%$, $\chi^2_{20} = 2242.25$ ($p = 0$)			

Figure S1A Forest plot of subgroup analysis of the uptake in the last flu season by year

region = 2			
Bauer, 2021	0.44	[0.37; 0.52]	4.3%
Brown, 2011	0.39	[0.38; 0.41]	4.4%
Daley, 2007	0.71	[0.68; 0.74]	4.4%
Gentile, 2015	0.73	[0.71; 0.75]	4.4%
Hemingway, 2004	0.18	[0.14; 0.23]	4.3%
Ma, 2006	0.38	[0.32; 0.44]	4.3%
Nowalk, 2005	0.43	[0.38; 0.48]	4.4%
Nowalk, 2007	0.45	[0.39; 0.51]	4.3%
Schuller, 2013	0.56	[0.56; 0.57]	4.4%
Shinall, 2007	0.42	[0.35; 0.49]	4.3%
Williams, 2021	0.76	[0.71; 0.81]	4.3%
**Random effects model**	**0.50**	**[0.38; 0.61]**	**47.9%**
Heterogeneity: $I^2 = 99\%$, $\chi^2_{10} = 836.66$ ($p < 0.01$)			
region = 3			
Bults, 2011	0.36	[0.34; 0.38]	4.4%
Chantler, 2007	0.11	[0.05; 0.20]	3.8%
**Random effects model**	**0.21**	**[0.06; 0.55]**	**8.2%**
Heterogeneity: $I^2 = 95\%$, $\chi^2_{1} = 18.62$ ($p < 0.01$)			
region = 1			
Chen, 2011	0.60	[0.59; 0.62]	4.4%
Chen, 2015	0.50	[0.47; 0.53]	4.4%
Han, 2022	0.44	[0.41; 0.46]	4.4%
He, 2015	0.48	[0.42; 0.53]	4.4%
Low, 2017	0.15	[0.11; 0.19]	4.3%
Thanee, 2021	0.09	[0.07; 0.11]	4.3%
Wu, 2020	0.30	[0.26; 0.35]	4.4%
Wu, 2023	0.33	[0.31; 0.36]	4.4%
Zhang, 2022	0.39	[0.38; 0.41]	4.4%
**Random effects model**	**0.34**	**[0.23; 0.48]**	**39.4%**
Heterogeneity: $I^2 = 99\%$, $\chi^2_{8} = 791.9$ ($p < 0.01$)			
region = 4			
Frawley, 2020	0.53	[0.50; 0.56]	4.4%
**Random effects model**	**0.41**	**[0.33; 0.50]**	**100.0%**
Heterogeneity: $I^2 = 99\%$, $\chi^2_{22} = 2442.02$ ($p = 0$)			

Figure S1B Forest plot of subgroup analysis of the uptake in the last flu season by region

Study	Proportion	95%-CI	Weight
**size = 1**			
Bauer, 2021	0.44	[0.37; 0.52]	4.3%
Chantler, 2007	0.11	[0.05; 0.20]	3.8%
Daley, 2007	0.71	[0.68; 0.74]	4.4%
He, 2015	0.48	[0.42; 0.53]	4.4%
Hemingway, 2004	0.18	[0.14; 0.23]	4.3%
Low, 2017	0.15	[0.11; 0.19]	4.3%
Ma, 2006	0.38	[0.32; 0.44]	4.3%
Nowalk, 2005	0.43	[0.38; 0.48]	4.4%
Nowalk, 2007	0.45	[0.39; 0.51]	4.3%
Shinall, 2007	0.42	[0.35; 0.49]	4.3%
Thanee, 2021	0.09	[0.07; 0.11]	4.3%
Williams, 2021	0.76	[0.71; 0.81]	4.3%
Wu, 2020	0.30	[0.26; 0.35]	4.4%
**Random effects model**	**0.35**	**[0.23; 0.49]**	**55.8%**
Heterogeneity: $I^2 = 99\%$, $\chi^2_{12} = 801.84$ ($p < 0.01$)			
**size = 2**			
Brown, 2011	0.39	[0.38; 0.41]	4.4%
Bults, 2011	0.36	[0.34; 0.38]	4.4%
Chen, 2011	0.60	[0.59; 0.62]	4.4%
Chen, 2015	0.50	[0.47; 0.53]	4.4%
Frawley, 2020	0.53	[0.50; 0.56]	4.4%
Gentile, 2015	0.73	[0.71; 0.75]	4.4%
Han, 2022	0.44	[0.41; 0.46]	4.4%
Schuller, 2013	0.56	[0.56; 0.57]	4.4%
Wu, 2023	0.33	[0.31; 0.36]	4.4%
Zhang, 2022	0.39	[0.38; 0.41]	4.4%
**Random effects model**	**0.48**	**[0.40; 0.57]**	**44.2%**
Heterogeneity: $I^2 = 99\%$, $\chi^2_{9} = 1533.42$ ($p = 0$)			
**Random effects model**	**0.41**	**[0.33; 0.50]**	**100.0%**
Heterogeneity: $I^2 = 99\%$, $\chi^2_{22} = 2442.02$ ($p = 0$)			

Figure S1C Forest plot of subgroup analysis of the uptake in the last flu season by sample size

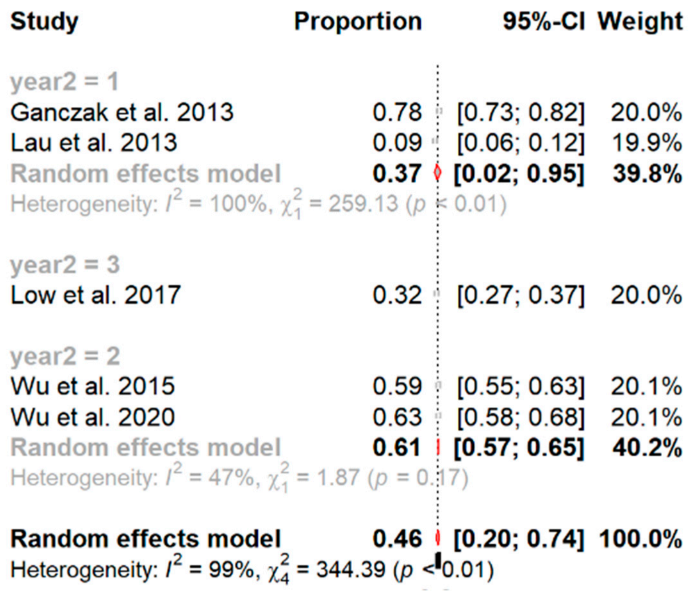

Figure S2A Forest plot of subgroup analysis of the uptake in lifetime by year

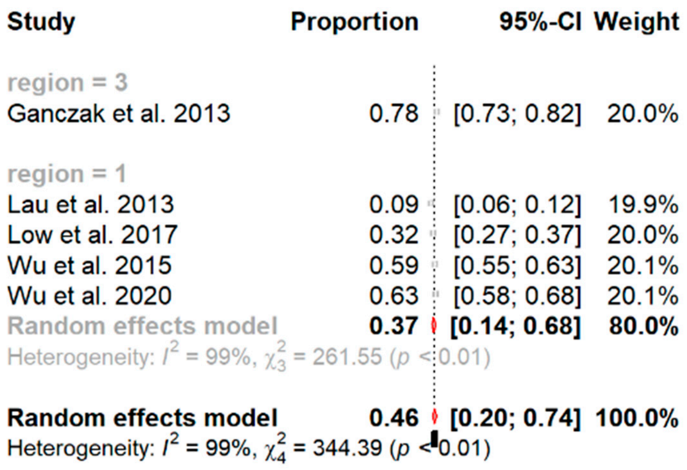

Figure S2B Forest plot of subgroup analysis of the uptake in lifetime by region

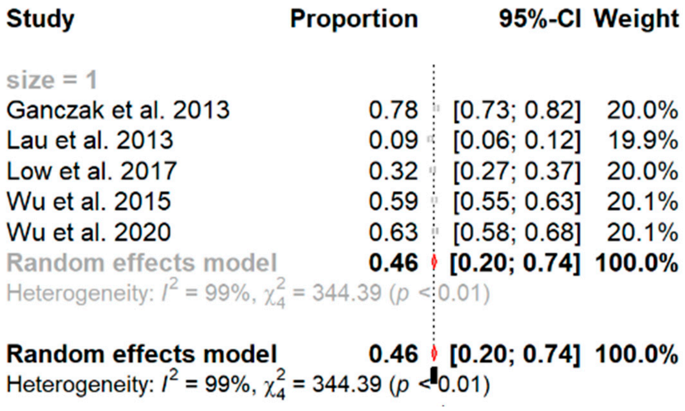

Figure S2C Forest plot of subgroup analysis of the uptake in lifetime by sample size

Study	Proportion	95%-CI	Weight
**year2 = 3**			
Bauer et al. 2021	0.71	[0.63; 0.78]	7.0%
Fisher et al. 2022	0.66	[0.59; 0.73]	7.1%
Lai et al. 2022	0.39	[0.37; 0.40]	7.2%
Schellenberg et al. 2023	0.88	[0.87; 0.89]	7.2%
Wei et al. 2021	0.57	[0.54; 0.59]	7.2%
Wu et al. 2023	0.40	[0.37; 0.43]	7.2%
**Random effects model**	**0.62**	**[0.44; 0.77]**	**43.0%**
Heterogeneity: $I^2 = 100\%$, $\chi^2_5 = 2905.62$ ($p = 0$)			
**year2 = 2**			
Chen et al. 2015	0.73	[0.70; 0.75]	7.2%
Choi et al. 2017	0.84	[0.80; 0.86]	7.2%
He et al. 2015	0.62	[0.57; 0.68]	7.1%
Lau et al. 2021	0.69	[0.65; 0.73]	7.2%
van Lier et al. 2017	0.15	[0.12; 0.19]	7.1%
Wu et al. 2020	0.49	[0.45; 0.54]	7.2%
**Random effects model**	**0.59**	**[0.36; 0.78]**	**43.0%**
Heterogeneity: $I^2 = 99\%$, $\chi^2_5 = 521.51$ ($p < 0.01$)			
**year2 = 1**			
Humiston et al. 2005	0.78	[0.70; 0.84]	7.0%
**year2 = 4**			
Williams et al. 2021	0.87	[0.82; 0.91]	7.0%
**Random effects model**	**0.64**	**[0.51; 0.75]**	**100.0%**
Heterogeneity: $I^2 = 100\%$, $\chi^2_{13} = 3619.22$ ($p = 0$)			

Figure S3A Forest plot of subgroup analysis of the parental acceptance by year

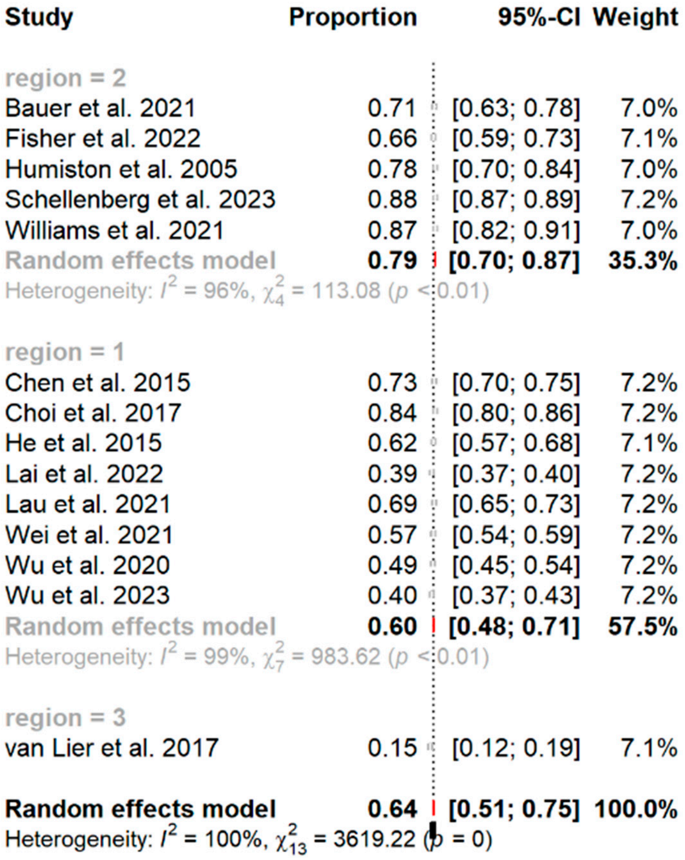

Figure S3B Forest plot of subgroup analysis of the parental acceptance by region

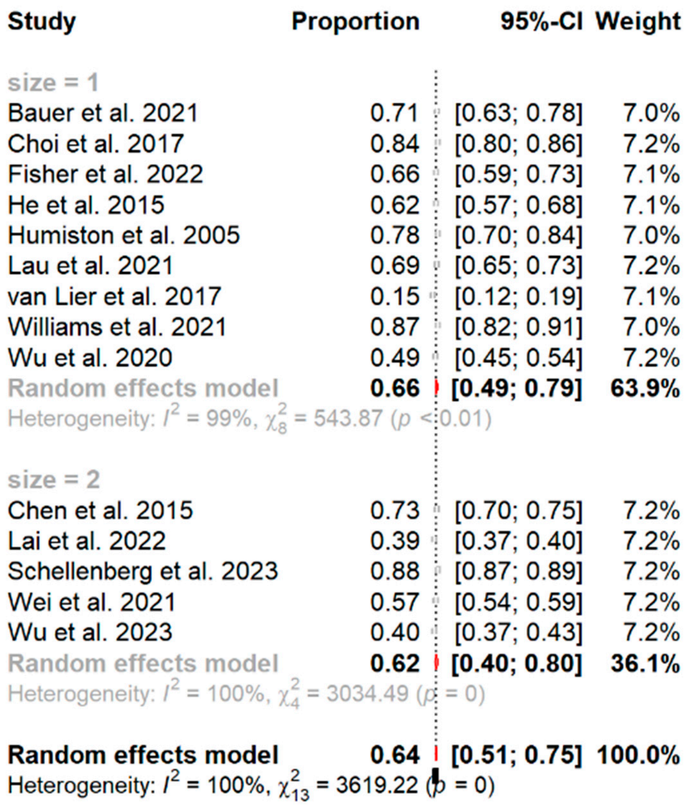

Figure S3C Forest plot of subgroup analysis of the parental acceptance by sample size

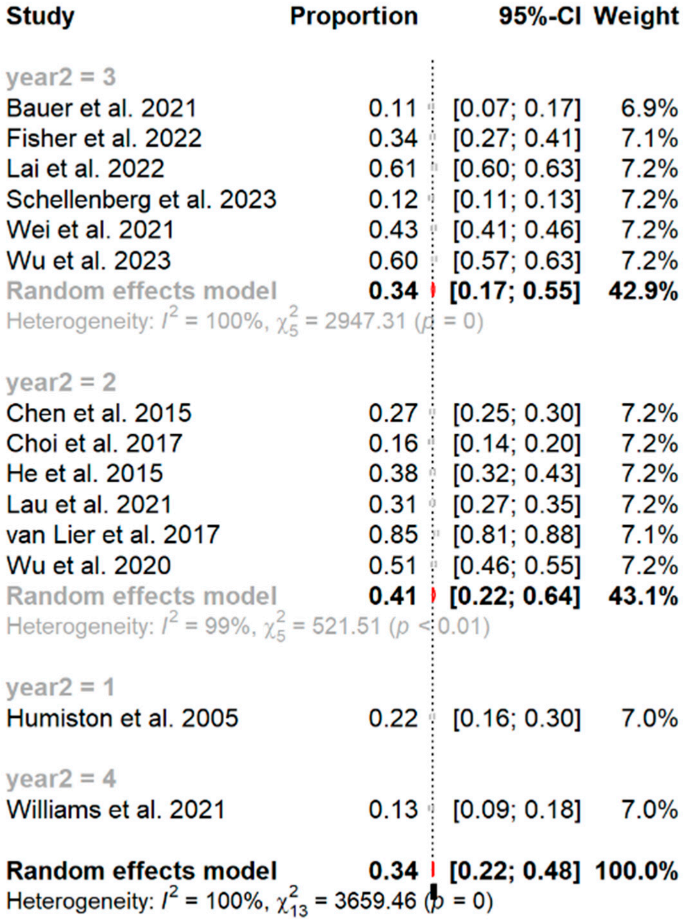

Figure S4A Forest plot of subgroup analysis of the parental hesitancy by year

Study	Proportion	95%-CI	Weight
**region = 2**			
Bauer et al. 2021	0.11	[0.07; 0.17]	6.9%
Fisher et al. 2022	0.34	[0.27; 0.41]	7.1%
Humiston et al. 2005	0.22	[0.16; 0.30]	7.0%
Schellenberg et al. 2023	0.12	[0.11; 0.13]	7.2%
Williams et al. 2021	0.13	[0.09; 0.18]	7.0%
**Random effects model**	**0.17**	**[0.11; 0.26]**	**35.3%**
Heterogeneity: $I^2 = 95\%$, $\chi^2_4 = 83.43$ ($p < 0.01$)			
**region = 1**			
Chen et al. 2015	0.27	[0.25; 0.30]	7.2%
Choi et al. 2017	0.16	[0.14; 0.20]	7.2%
He et al. 2015	0.38	[0.32; 0.43]	7.2%
Lai et al. 2022	0.61	[0.60; 0.63]	7.2%
Lau et al. 2021	0.31	[0.27; 0.35]	7.2%
Wei et al. 2021	0.43	[0.41; 0.46]	7.2%
Wu et al. 2020	0.51	[0.46; 0.55]	7.2%
Wu et al. 2023	0.60	[0.57; 0.63]	7.2%
**Random effects model**	**0.40**	**[0.29; 0.52]**	**57.6%**
Heterogeneity: $I^2 = 99\%$, $\chi^2_7 = 983.62$ ($p < 0.01$)			
**region = 3**			
van Lier et al. 2017	0.85	[0.81; 0.88]	7.1%
**Random effects model**	**0.34**	**[0.22; 0.48]**	**100.0%**
Heterogeneity: $I^2 = 100\%$, $\chi^2_{13} = 3659.46$ ($p = 0$)			

Figure S4B Forest plot of subgroup analysis of the parental hesitancy by region

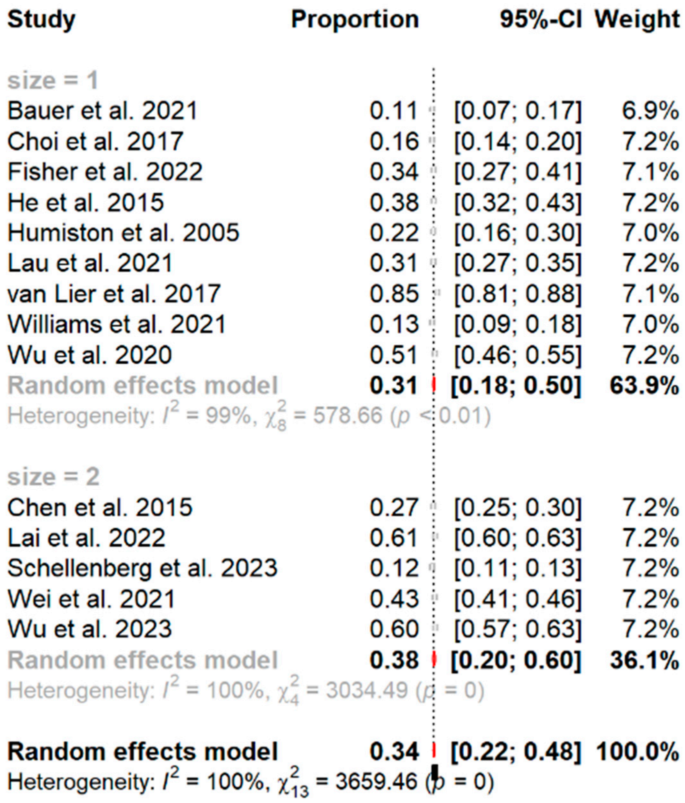

Figure S4C Forest plot of subgroup analysis of the parental hesitancy by sample size
